# Diagnostic performance of peripheral nerve palpation compared with ultrasonography in leprosy neuropathy: A prospective real-world clinical evaluation

**DOI:** 10.1371/journal.pntd.0014234

**Published:** 2026-04-21

**Authors:** Jaqueline da Silva Mendes, Thiago Montenegro da Silva, Patricia Tavares Cruz, Amanda Gabrielle dos Santos Cordeiro, Raquel da Mata Serique, Marcelo Ribeiro Alves, Flavio Alves Lara, Carolina Talhari, Hélio Amante Miot, Sinésio Talhari

**Affiliations:** 1 Programa de Pós-Graduação em Ciências Aplicadas à Dermatologia, Universidade do Estado do Amazonas, Manaus, Brazil; 2 Fundação Hospitalar Alfredo da Matta, Manaus, Brazil; 3 Instituto Nacional de Infectologia (IPEC) Fundação Oswaldo Cruz, Rio de Janeiro, Brazil; 4 Lab. de Microbiologia Celular, Instituto Oswaldo Cruz, Fundação Oswaldo Cruz, Rio de Janeiro, Brazil; 5 Departamento de Dermatologia e Radioterapia da FMB-UNESP, Botucatu, Brazil; Mahidol Univ, Fac Trop Med, THAILAND

## Abstract

**Background:**

Leprosy frequently causes peripheral neuropathy, and nerve palpation remains a cornerstone of diagnosis in many endemic settings despite its subjectivity and limited reproducibility. High-resolution ultrasonography has emerged as a sensitive, low-cost imaging tool to detect peripheral nerve involvement. We aimed to compare the diagnostic performance and interobserver agreement of peripheral nerve palpation versus ultrasonography for detecting leprosy neuropathy, with particular focus on pure neural forms.

**Methodology/Principal findings:**

In this cross-sectional study, 29 newly diagnosed, treatment-naïve patients with clinical neuropathy underwent standardized palpation of the radial, ulnar, fibular, and posterior tibial nerves by three experienced examiners (dermatologist, orthopedist, physiotherapist). Ultrasonographic cross-sectional area was used as a comparative reference method. Overall, 232 nerves were evaluated, and ultrasonography identified nerve thickening in 26%. Interobserver agreement for nerve thickening on palpation was 60% (95% CI: 54–65%). Using ultrasonography as reference, palpation sensitivity ranged from 26% to 34%, specificity from 68% to 81%, and accuracy from 56% to 67%. Among individual nerves, the posterior tibial nerve showed the highest palpation sensitivity (44%), whereas thickening of the radial nerve was rarely detected (3%). Bilateral asymmetry (>2.5 mm²) was observed in 13% of nerves that were considered clinically normal on examination, and median nerve enlargement (at ultrasonography) was present in 45% of the participants. In patients with pure neural leprosy (62% of the sample), palpation sensitivity was 43%, resulting in frequent misclassification of operational forms.

**Conclusions/Significance:**

Peripheral nerve palpation showed low sensitivity and only moderate specificity when compared with ultrasonography, with poor interobserver agreement even among experienced clinicians. These findings support the consideration of high-resolution ultrasonography as a complementary tool in diagnostic algorithms, particularly in pure neural cases, where reliance on palpation alone may lead to underdiagnosis and misclassification.

## Author summary

Leprosy can damage the nerves in the arms and legs, leading to pain, numbness, weakness, and lifelong disability if it is not recognized early. In many endemic regions, we still rely mainly on feeling the nerves with our fingers to decide whether they are thickened. This method is simple and inexpensive, but it is subjective and may miss early or subtle nerve involvement. In this study, we compared routine nerve palpation performed by three experienced clinicians with high-resolution nerve ultrasound imaging in patients with leprosy-related nerve damage, including those without skin lesions. We found that clinical palpation detected only a minority of the nerves that were enlarged on ultrasound and that agreement between examiners was limited. These shortcomings frequently led to mistakes in classifying patients for treatment. Our findings suggest that incorporating nerve ultrasound, including portable devices, into the evaluation of patients with suspected leprosy neuropathy could improve early diagnosis, guide correct treatment decisions, and help prevent disability, particularly in people who have nerve symptoms but no visible skin lesions.

## Introduction

Leprosy is a chronic infectious disease caused by *Mycobacterium leprae* and *Mycobacterium lepromatosis*, which primarily affects the skin and peripheral nerves. Due to its involvement of the peripheral nervous system, clinical manifestations range from autonomic changes to severe disabilities and disfigurement, accompanied by loss of productivity and profound psychosocial consequence [1–3].

The bacilli infect cutaneous macrophages and adipocytes, as well as Schwann cells, leading to inflammation and progressive nerve damage that compromises sensory, motor, and autonomic functions [2,3]. Sensory deficits vary from loss of sensation to hypoesthesia, while motor dysfunction may progress from weakness to irreversible paralysis [4,5]. Early diagnosis and prompt multidrug therapy are essential to prevent disability, which is often irreversible; however, the early detection of neural manifestations remains a challenge [6].

Clinical examinations such as sensory and strength testing, and particularly nerve palpation for the detection of thickening, have been employed for decades as low-cost tools for neural assessment in leprosy. According to the Brazilian Ministry of Health (BMH), clinical diagnosis of neural leprosy should be early and accurate, based on the identification of cardinal signs of the disease, including peripheral nerve thickening associated with sensory, motor, or autonomic impairment. This requires the training of health professionals in nerve palpation and the performance of simple neurological tests. National guidelines also state that the involvement of more than one nerve, with loss or reduction of sensitivity, operationally defines multibacillary (MB) disease. In contrast, WHO guidelines recommend that even the involvement of a single nerve is sufficient for this classification, which diverges from Brazilian recommendations [7–9]. Meanwhile, the differential diagnosis of pure neural leprosy (PNL) among other polyneuropathies remains a challenge, especially in leprosy-endemic areas.

The palpation of peripheral nerves is highly subjective, examiner-dependent, and prone to considerable inter-observer variability [10]. Even experienced professionals may struggle to detect subtle nerve thickness, particularly in obese individuals or those with greater muscle mass. This becomes especially critical in cases of PNL, where no skin lesions are present, the slit skin smear (SSS) test is negative, and the diagnosis relies solely on neurological semiology while excluding other neuropathies. It is estimated that up to 10% of reported leprosy cases in Brazil represent pure neural forms [6]. Moreover, the diagnosis of leprosy is often performed by a range of healthcare professionals.

Anti–phenolic glycolipid-I lateral flow assay (ML Flow), while not routinely recommended for leprosy diagnosis, can serve as an auxiliary tool in research and epidemiological contexts, particularly in endemic areas. This serological test can help identify individuals with subclinical or early *M. leprae* infection, especially those presenting neural symptoms but no cutaneous lesions. Anti-PGL-1 seropositivity increases the likelihood that neuropathy is attributable to leprosy rather than other etiologies. In research settings, this marker supports risk stratification and diagnostic refinement for pure neural forms. Nevertheless, its sensitivity remains limited in PB and pure neural cases, and results must be interpreted in conjunction with clinical, imaging, and, when available, molecular data [11,12].

Although electrophysiological studies, such as nerve conduction tests, provide objective data on nerve function, they are often unavailable in many clinical settings that manage leprosy and do not always identify the exact location or extent of nerve damage [12]. Even in referral centers, access to this examination is limited, as few institutions have the necessary equipment, and many professionals who perform these tests, particularly in nonendemic regions, lack specific training and experience in leprosy.

High-frequency ultrasonography has emerged as a valuable, non-invasive tool for the evaluation of peripheral nerves in leprosy. Its cost-effectiveness and ability to visualize nerve structures as small as 1 mm make it particularly suitable for the early diagnosis of leprosy neuropathy. This method allows detailed assessment of cross-sectional area (CSA), echotexture, and vascularization, enabling the detection of characteristic findings such as fusiform nerve thickening, increased blood flow, and abscess formation [11,13–16]. Importantly, ultrasonography has proven especially useful for diagnosing PNL, in which dermatological signs are absent, and for identifying typical patterns of neural involvement, such as asymmetry and focality [17].

Furthermore, ultrasonography can be used to monitor treatment response in leprosy patients, correlating functional improvement with changes in imaging findings. It can also guide procedures such as nerve biopsies or localized injectable treatments, thereby improving diagnostic and therapeutic precision [18,19].

Despite these advantages, ultrasonography remains underutilized in leprosy care, even in referral centers, where nerve palpation continues to be the predominant examination technique, and only a few studies have validated its role as a clinical diagnostic adjunct. Studies assessing agreement between ultrasonography and palpation, as well as inter-observer reliability, remain scarce [20].

This study aimed to evaluate the diagnostic performance of peripheral nerve palpation in leprosy, using ultrasonography as a pragmatic comparative reference method, and to compare inter-examiner agreement among three experienced professionals performing clinical palpation.

## Methods

### Ethics statement

The study was approved by the FUAM Ethics Committee (CAAE: 81197024.80000.0002). In addition, CAAE nº 80959224.40002.0002 was incorporated, corresponding to the integration of another project related to the topic, ensuring ethical compliance of all proposed procedures. All participants provided written informed consent.

### Study design

This was a cross-sectional study conducted at Fundação Hospitalar Alfredo da Matta (FUHAM), a leprosy referral center in Manaus, Amazonas state, Brazil, between June 2024 and September 2025.

Twenty-nine treatment-naïve patients diagnosed with leprosy with concomitant neuropathy, defined as ≥1 enlarged peripheral nerve on physical examination, and negative Slit-skin smear bacilloscopy, were enrolled. The diagnosis of PNL, BB, or BT was established before enrollment and was independent of the nerve palpation and ultrasonography assessments performed for this study. Notably, PNL cases were close contacts of individuals with leprosy and originated from the same endemic area; these epidemiological factors were explicitly considered during case definition to strengthen the diagnosis of PNL, which is inherently challenging. Females comprised 62% of the cohort, PNL was the most frequent clinical form (62%), and ML Flow was positive in 9 patients (31%) (Table 1).

**Table 1:** Main clinical and demographic data of the sample (*n* = 29).

Variables	Values
Age (years), mean (SD)	43.5 (13.5)
Sex, n (%)	
Female	18 (62%)
Male	11 (38%)
Education, n (%)	
Primary school	3 (10%)
High school	19 (66%)
Higher education	7 (24%)
Clinical Spectrum, n (%)	
Borderline tuberculosis (BT)	9 (31%)
Bordeline borderline (BB) Pure Neural Leprosy (PNL)	2 (7%) 18 (62%)
Self-declared race, n (%)	
Mixed-race/ Amerindian	24 (83%)
White	4 (14%)
Black	1 (3%)
Disability grade, n (%)	
G0D	3 (10%)
G1D	15 (52%)
G2D	11 (38%)
ML Flow positive, n (%)	9 (31%)
BMI (kg/m²), mean (SD)	27.8 (6.4)
Nerves thickened by ultrasonography, n (%)*	
Radial	10 (17%)
Ulnar	18 (31%)
Posterior tibial	16 (28%)
Fibular	21 (36%)
Median	21 (36%)
Thickened nerves per participant, median (p25-p75)	2 (1-3)

SD: standard deviation; NA: not available; PNL: pure neural leprosy; BMI: body mass index; BT: borderline tuberculoid; BB: borderline. * n = 58 for each nerve.

All patients underwent a comprehensive clinical and physical investigation, including nerve palpation, which was performed independently by three experienced health professionals. Further investigation included disability grade (GD) assessment, ML Flow, and peripheral nerve ultrasonography. Patient height and weight were recorded for the calculation of body mass index (BMI).

### Leprosy and PNL diagnosis

In the present study, leprosy was defined according to Brazilian Ministry of Health (BMH) criteria as the presence of ≥1 cardinal sign: (i) skin lesion(s) or area(s) with impaired thermal, pain, or tactile sensation; (ii) thickening of a peripheral nerve with corresponding sensory, motor, and/or autonomic dysfunction. Additionally, PNL was defined per the same criteria as an exclusively neural presentation with no cutaneous lesions; clinically, diagnosis required the second cardinal sign, thickening of a peripheral nerve accompanied by sensory, motor, and/or autonomic impairment in the nerve’s distribution [6].

### Nerve palpation

Nerve palpation was performed according to the guidelines of the BMH [5,6], using gentle maneuvers to minimize patient discomfort. The course of each nerve was traced with the pulp of the examiner’s second and third fingers, and any reports of pain or paresthesia (e.g., electric shock–like sensations) were documented.

Three experienced health professionals: a dermatologist, an orthopedist, and a physiotherapist, each with formal training in leprosy neuropathy assessment and serving as attending staff at the referral center, independently examined every nerve. To minimize examiner-related variability, all three applied the same standardized palpation protocol, using identical hand positioning and digital rolling along the nerve course with graded pressure and segment-by-segment evaluation. They assessed nerve thickness, contour, adhesions to deep planes, and nodularity uniformly, and the contralateral homonymous nerve was used as an internal comparator for bilateral reference [5,6].

Ulnar nerves were palpated in the epitrochlear groove or proximally, with the elbow flexed at 90°. Radial nerves were palpated in the humeral spiral groove (mid-arm region), with the elbow flexed and the patient’s hand resting on the examiner’s hand. Common fibular nerves were palpated at the posterior junction of the fibular head and shaft, with the patient seated and legs freely dangling. Posterior tibial nerves were palpated posterior and inferior to the medial malleolus, with the patient seated and the leg hanging freely at a 90° angle between the foot and the leg. Following evaluation, each nerve was classified by the examiners as normal (palpable without thickening), thickened, or not palpable (S1 Fig) [5].

To maintain impartiality and reduce observer bias, all assessments were individually recorded on standardized forms using a blinded methodology, in which the examiners had no access to each other’s findings during the evaluation process.

### Peripheral nerve ultrasonography

Peripheral nerves (median, radial, ulnar, fibular, and posterior tibial) were evaluated using a Logiq P9 system (GE HealthCare), equipped with a 13-MHz linear transducer (Probe L6-12). The pulse repetition frequency was set at 1 kHz, and the filter bandwidth was adjusted to 50 Hz. Ultrasonographic CSA was used as a comparative reference method in this study. Threshold values for nerve thickening in adults were defined as follows: median nerve >10.0 mm², radial nerve >8.0 mm², ulnar nerve >9.4 mm², fibular nerve >11.7 mm², and posterior tibial nerve >9.9 mm² [15,21]. All ultrasonographic examinations were performed by the same professional, an experienced radiologist with over 10 years of practice and specific training in musculoskeletal and peripheral nerve ultrasonography, who was blinded to the clinical palpation results.

The radial nerve was examined with the patient in the supine position, elbow slightly flexed, and posterior arm exposed. The radial nerve was identified just beneath the deltoid muscle, in the radial groove. The transducer was positioned perpendicular to the nerve axis, allowing visualization of continuity and possible alterations along its course (such as compressions, focal thickenings, or discontinuities).

The ulnar nerve was examined at the medial epicondyle and 4 cm above, with the patient’s arm abducted and elbow flexed less than 90°.

The posterior tibial nerve was examined with the lower limb in slight external rotation to facilitate access to the medial ankle. The probe was placed transversely, perpendicular to the nerve, 3 cm above and 5 cm posterior to the medial malleolus.

The common fibular nerve was assessed with the patient in lateral decubitus and knees fully extended. The probe was positioned transversely over the fibular head, perpendicular to the nerve.

As shown in S2 Fig, the ultrasonographic examination technique for nerve evaluation is demonstrated.

The median nerve, despite not being palpable, was assessed in this study by ultrasonography at the wrist and 4 cm proximally, with the patient positioned supine and arms alongside the body in supination [16].

Afterward, the CSA of each nerve was immediately traced within the hyperechoic border of the epineurium. Nerve enlargement was defined a priori using published normative reference values (adult populations) for each specific nerve, adopting the corresponding upper reference limit (e.g., 95th percentile/upper 95% CI) as the threshold for abnormality [15]. No local control group was used. Patients with CSA values exceeding the predefined reference limit for that nerve were classified as having nerve enlargement for subsequent analyses.

### Statistical analysis

Data were described as absolute numbers and percentages, or as mean and standard deviation (SD) if the normality was confirmed by the Shapiro-Wilk test, otherwise, as median and quartiles (p25-p75) [22]. To assess the reliability of neural palpation, interobserver agreement among the three independent clinicians for classifying nerves as “normal,” “thickened,” or “not palpable” was calculated.

The diagnostic performance of palpation compared with ultrasonography (reference method) was assessed by calculating accuracy (overall correct classification), sensitivity, and specificity for detecting nerve thickening, both for each nerve (radial, ulnar, fibular, posterior tibial) and for the pooled dataset.

Diagnostic performance metrics were calculated according to established definitions, using ultrasonography as the comparative reference method [23–25]. Sensitivity was defined as the proportion of nerves identified as thickened by ultrasonography that were correctly classified as “thickened” by palpation. Specificity was pragmatically defined as the proportion of nerves classified as normal by ultrasonography and correctly identified as “normal” or “not palpable” by palpation. Accuracy was defined as the proportion of all ultrasonographically evaluated nerves (thickened or normal) that were correctly classified by palpation.

Diagnostic performance metrics (sensitivity, specificity, and accuracy) evaluate the ability of palpation to detect ultrasonographically defined nerve enlargement, whereas agreement metrics (Fleiss’ and Cohen’s kappa) assess concordance between methods beyond chance. These measures address complementary but distinct aspects of test performance [26].

The correlation between the clinical identification rate of thickened nerves and BMI was assessed using Spearman’s rho coefficient [27]. Comparisons across multiple groups were performed using Cochran’s Q test, and subgroup comparisons for the number of affected nerves were analyzed using the Student’s *t*-test or Mann-Whitney, if appropriate.

A (two-tailed) *p*-value < 0.05 was considered statistically significant for all comparisons [28]. All analyses were performed using IBM SPSS Statistics, version 31.

Sample size estimation was based on an assumed inter-examiner agreement of kappa = 0.45, a 95% confidence interval with a half-width of 0.15, a binary outcome, and three examiners, with a 30% prevalence of thickened nerves, α = 5%, and 80% power. This required the evaluation of a minimum of 180 nerves [29].

## Results

In total, 232 nerves were clinically examined, of which 65 (28%) showed thickening on ultrasonography. In disability assessment, most participants presented G1D (52%), followed by G2D (38%). Seven participants (24%) presented with all nerves palpable and sonographically normal, five of whom had the pure neural form of the disease; three of these had G2D (Table 1). There was no significant difference (*p* = 0.87) in the median (p25-p75) number of thickened palpable nerves between patients with PNL and those with cutaneous lesions: 2 (1–4) vs. 2 (1–2). Likewise, no difference was observed between anti-PGL-1–positive and –negative patients (*p* = 0.07)

Neural thickening was identified in 29%, 23%, and 31% of clinical examinations performed by the three evaluators, whereas nerves were not identified in 32%, 30%, and 30% of cases, respectively. The radial nerve was not identified in 85%, 91%, and 78% of assessments. Among the 50 nerves (22%) not palpated by any of the three evaluators, 16 (32%) were found to be thickened on ultrasonography.

**Fig 1:**
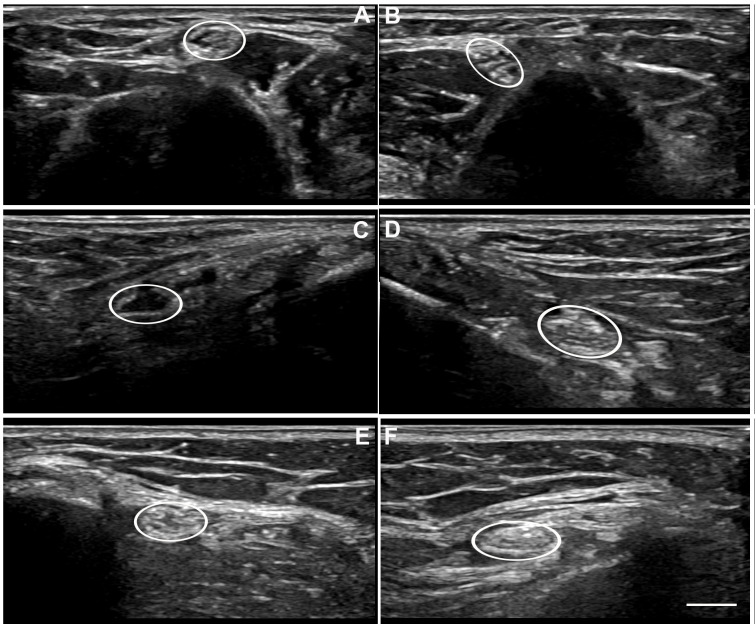
Representative ultrasonographic images of the nerves. Transverse ultrasonographic images of a normal ulnar nerve **(A)**, a thickened ulnar nerve **(B)**, a normal tibial nerve **(C)**, a thickened tibial nerve **(D)**, a normal common fibular nerve **(E)**, and a thickened common fibular nerve **(F)**, acquired with an ultrasound device Logiq P9. Scale bar: 0.5 mm.

Overall agreement among the three evaluators regarding the detection of neural thickening was 60% (95% CI: 54–65%), with no significant difference between evaluator pairs (*p* = 0.06). Table 2 presents the pairwise agreement values for each nerve studied. The kappa coefficient indicated poor overall agreement and no agreement for the radial nerve. When analyses involving the radial nerve were excluded, the overall agreement of the evaluators was 49% (95% CI: 41–55%). Fig 1 shows representative images obtained with the ultrasound device.

**Table 2:** Agreement in the diagnosis of neural thickening according to evaluators and nerve groups.

Variables	Agreement	95% CI	Kappa coefficient	p-value
Overall agreement (*n* = 232)	139 (59.9%)	53.5% – 65.1%	0.33	<0.01
Av1 × Av2	172 (74.1%)	68.5% – 78.9%	0.38	<0.01
Av1 × Av3	172 (74.1%)	68.5% – 78.9%	0.38	<0.01
Av2 × Av3	166 (71.6%)	65.5% – 75.9%	0.28	<0.01
By nerve group (*n* = 58):				
Radial	54 (93.1%)	86.2% – 98.3%	-0.02	0.58
Ulnar	30 (51.7%)	37.9% – 62.1%	0.26	<0.01
Posterior tibial	27 (46.6%)	34.5% – 56.9%	0.26	<0.01
Fibular	28 (48.3%)	36.2% – 58.6%	0.28	<0.01

Av1, Av2, Av3: Health professionals experienced in neural palpation for leprosy; 95% CI: 95% confidence interval.

Table 3 summarizes the agreement between clinical palpation and ultrasonography in identifying nerve thickening. When palpation findings were compared with ultrasonographic results, the overall diagnostic accuracy ranged from 56% to 67% across evaluators. However, kappa coefficients indicated poor agreement beyond chance (<0.20), despite moderate overall percentage agreement, highlighting the influence of prevalence and marginal distributions on agreement metrics. When radial nerve assessments were excluded, diagnostic accuracy ranged from 48% to 63% across evaluators.

**Table 3:** Sensitivity, specificity, and accuracy of the diagnosis of neural thickening by ultrasonography and evaluators (*n* = 232).

Variables	Sensitivity	Specificity	Accuracy	Kappa coefficient	p-value
Overall examination^a^					
Av1	33.8% (22.1% – 45.6%)	73.1% (66.3% – 79.8%)	62.1% (54.1% – 70.0%)	0.07	0.30
Av2	32.3% (20.7% – 43.9%)	80.8% (74.8% – 86.9%)	67.2% (59.8% – 74.6%)	0.14	0.03
Av3	26.2% (15.3% – 37.0%)	67.7% (60.5% – 74.8%)	56.0% (47.5% – 64.6%)	-0.03	0.45
Radial nerve^b^	3.3% (0% – 10.0%)	97.9% (95.6% – 100.0%)	81.6% (75.2% – 88.0%)	0.02	0.68
Ulnar nerve^b^	33.3% (20.5% – 46.2%)	69.2% (60.8% – 77.5%)	58.0% (48.4% – 67.7%)	0.03	0.74
Posterior tibial nerve^b^	43.8% (29.3% – 58.2%)	61.9% (53.3% – 70.5%)	56.9% (47.1% – 66.7%)	0.05	0.50
Fibular nerve^b^	31.7% (20.0% – 43.5%)	61.3% (52.1% – 70.4%)	50.6% (40.1% – 61.1%)	-0.07	0.36

^a^ 232 nerves; ^b^ 174 evaluations (Av1, Av2, and Av3) in 58 nerves;

Av1, Av2, Av3: Health professionals experienced in neural palpation in leprosy cases.

When nerves were analyzed individually, the highest sensitivity was observed for the posterior tibial nerve (44%), whereas radial nerve palpation showed a sensitivity of only 3%. Patient BMI did not correlate with the rate of thickened nerve identification by palpation among evaluators 1, 2, and 3 (*rho* = –0.28, –0.34, and –0.08; p > 0.13).

Among thickened nerves, 56% were unilateral. Of the 71 pairs of normal nerves, nine (12.7%) showed bilateral variation >2.5 mm², compared with 21 (84%) of the 25 pairs with unilateral thickening and seven (35%) of the 20 pairs with bilateral thickening.

When the 18 patients with PNL (144 nerves) were analyzed, palpation sensitivity was 43% (95% CI: 28%–58%), specificity 72% (95% CI: 63–80%), and accuracy 63% (95% CI: 53–73%). The ML Flow test, used descriptively in this sample, was positive in 31% of cases and did not appear to aid in screening these patients. Evaluators failed to detect 57% of thickened nerves by palpation in this subgroup.

The median nerve was thickened in 13 cases (45%), including eight (44%) among patients with PNL. In one patient without skin lesions but with neurological abnormalities (G2D), only the median nerves were bilaterally thickened, which operationally would classify the case as multibacillary (MB). In this instance, two evaluators failed to identify any other thickened peripheral nerves, while the third misclassified two nerves as falsely thickened.

Among the 18 PNL cases, six (33%) presented fewer than two thickened nerves and should have been treated as paucibacillary (PB). Misclassification based on nerve palpation occurred in 100%, 33%, and 50% of evaluations (MB diagnosed in PB cases), and in 17%, 25%, and 25% (PB diagnosed in MB cases).

In four patients with PNL, ultrasonography detected no thickening of any assessed peripheral nerve, including the median nerve. Three of these had positive ML Flow, and in two cases bilateral asymmetry >2.5 mm² was observed; in one of them (with G2D), asymmetry involved two nerves, which could have altered the operational classification.

## Discussion

Leprosy is a disease with a long incubation period, an indolent course, and multiple dermatological and neurological manifestations. These features hinder and delay diagnosis in primary care, leading to higher disability grades, morbidity, and transmission risk. This study included only cases with negative SSS test and neurological complaints, which highlights the relevance of neurological examination in defining the diagnosis of leprosy and simulates the clinical challenges faced in primary health care units. However, our results demonstrate that peripheral nerve palpation has limited sensitivity for detecting structural nerve enlargement when compared with ultrasonography, even when performed by experienced examiners. Although overall agreement appeared moderate, kappa statistics indicated poor concordance beyond chance, reinforcing the variability and subjectivity inherent to clinical palpation. These findings suggest that palpation alone may be insufficient for reliable detection of neural involvement, particularly in early or pure neural presentations. The low sensitivity and accuracy observed in this sample are consistent with previous studies documenting the subjective nature of manual nerve evaluation [13,30–32].

In this cross-sectional study, only 30% of thickened nerves were detected by experienced evaluators. The low sensitivity of palpation compared with ultrasonography is consistent with studies showing that ultrasonography detects thickening, asymmetry, and focality more objectively than clinical examination alone, and even before abnormalities appear on electroneuromyography [20,33]. In series with neuropathological and electrophysiological correlation, ultrasonography outperformed clinical examination in detecting nerve thickening and additionally characterized fascicular architecture and vascularization, providing further information for management [34]. Importantly, BMI did not correlate with difficulty in detecting thickened nerves, suggesting that confounding factors in this diagnostic method are more complex and less understood than the simple challenge of palpating neural fascicles beneath thick layers of subcutaneous fat.

An Indian study that identified 47% of nerves as thickened in 40 patients with leprosy found that examiners detected only 20% of these by palpation (sensitivity of 42%), with greater agreement for ulnar and fibular nerve thickening [20]. Beyond underdiagnosis, the authors also highlighted the risk of misclassification (PB vs. MB), since fewer affected nerves were identified. Even with evaluators experienced in leprosy, the low inter-examiner agreement, assessed for the first time in our study, did not mitigate the poor sensitivity and specificity of clinical evaluation. Collectively, these findings emphasize the importance of training in neurological examination (not limited to nerve palpation) for primary care, as well as the relevance of ultrasonographic support in referral centers to clarify doubtful cases.

To date, no mass screening tests are available that can establish a diagnosis of leprosy across all its clinical forms. According to the BMH, leprosy is defined when one or more of the following are present: cutaneous lesion or area with altered thermal, pain, or tactile sensation, and peripheral nerve thickening associated with sensory, motor, or autonomic changes. This clinical approach may be sufficient in at-risk individuals (e.g., in endemic areas) [6]. While this recommendation is justified by the epidemiological situation, it risks overdiagnosis of leprosy in conditions that also affect peripheral nerves, such as carpal tunnel syndrome, compressive radiculopathy, diabetes mellitus, alcoholism, pesticide exposure, nutritional deficiency, renal failure, hypothyroidism, traumatic/repetitive strain neuropathy, among others [35]. Conversely, our results highlight the limitations of nerve palpation as a reliable diagnostic tool for leprosy neuropathy, even when performed by trained clinicians. Another challenge lies in professional training: few patients present with pure neural forms, and few clinicians have the expertise to teach this skill. Identifying peripheral nerves is often difficult, and confirming whether they are thickened can be even more challenging in practice.

The diagnosis of leprosy is not made exclusively by physicians worldwide; in many endemic regions, nurses, community health agents, and mid-level technical health workers play a pivotal role in case detection and surveillance within public health systems. This decentralized diagnostic approach reflects both the scarcity of dermatologists and leprologists in primary-care settings and the community-based nature of leprosy control programs recommended by WHO. In countries such as Brazil, India, and several African nations, national control strategies explicitly authorize and train non-physician health workers to recognize the cardinal signs of leprosy: hypopigmented or erythematous anesthetic skin lesions, thickened peripheral nerves, and the presence of acid-fast bacilli on slit-skin smears. The diagnostic accuracy of non-physician health workers in leprosy remains a matter of debate, particularly in atypical or subtle presentations, such as those characterized by diffuse cutaneous infiltration or pure neural involvement [36,37].

Because palpation has low sensitivity and specificity, the Brazilian Ministry of Health recommends comparison with the contralateral limb, that is, palpation of the ulnar, fibular, posterior tibial, and even radial nerves bilaterally. However, asymmetry up to 2.5 mm², considered normal in the general population, may compromise even ultrasonographic diagnosis [17]. In this sample, 13% of nerve pairs classified as normal on ultrasonography exceeded this threshold. Thus, rigorous clinical evaluation, ultrasonography, and probably electroneuromyography are important for classification in cases with significant lateral asymmetry. It is also important to emphasize that diagnostic errors have major implications: patients may receive 6 or 12 months of MDT unnecessarily, be exposed to potential drug-related adverse events, undergo contact examination and laboratory testing, and experience stigma, all of which generate additional costs for both patients and health services.

The radial nerves proved difficult to assess clinically. In this sample, it was identified as thickened in only 17% of cases, but clinicians detected thickening in just 3.3%, undermining its usefulness for systematic screening of leprosy neuropathy. Despite their inclusion in the Brazilian simplified neurological evaluation guidelines [6], the radial nerves were identified by examiners in fewer than 23% of palpation attempts, with clearly insufficient performance for screening purposes. Even with ultrasonography, reliable visualization and measurement of the radial nerve remain limited, partly due to its deep anatomical position and individual morphological variability. This combination of low clinical detectability and technical limitations in ultrasonography calls into question the practical utility of routine radial nerve assessment in diagnostic protocols. Ulnar, posterior tibial, and fibular nerves, although more accessible, also demonstrated sensitivities below 45% and specificities below 70%, representing unsatisfactory performance for leprosy neuropathy screening. In this context, standard inclusion of radial nerve palpation could be reconsidered in future guideline updates, prioritizing more accessible and clinically relevant nerves such as ulnar, fibular, and posterior tibial, which show higher prevalence of involvement and better diagnostic accuracy.

The discordance between palpation and ultrasonography findings in this study highlights the urgent need for complementary diagnostic approaches with greater sensitivity and specificity in leprosy. The ML Flow test did not appear to aid in screening these patients, particularly PNL cases, which require heightened clinical suspicion and integration of neurological examination and epidemiological data. These findings support the adoption of integrated diagnostic algorithms that incorporate ultrasonography and serological tests to reduce misclassification, especially in pure neural cases with more than two thickened nerves and a negative SSS test (classified as MB by the BMH), and in single-nerve involvement, where Brazilian guidelines recommend PB classification [6]. This study identified errors in the operational classification of PNL cases, both PB and MB, when based solely on clinical palpation. Nerve biopsy may also play a role in diagnosis and classification; however, it carries the risk of iatrogenic nerve injury, and biopsies of sensory nerves, such as the sural or superficial radial branch, are often histologically normal in pure neural, usually PB cases [31,34].

In this study, considering PNL cases, only 43% of thickened nerves would have been detected by palpation, potentially compromising both diagnosis and operational classification that guide treatment. Despite higher specificity, 28% of ultrasonographically normal nerves in PNL patients were classified as thickened by examiners, which in endemic areas could lead to overdiagnosis in patients without leprosy but with peripheral neurological complaints, resulting in inappropriate or unnecessary treatment.

The use of ultrasound devices, including portable systems such as the one employed in this study, represents an essential and low-cost tool for the accurate evaluation of neural thickening, reinforcing its growing role in leprosy practice, as demonstrated in numerous reports over the past decade [10,13,16,38]. Recent advances in ultrasonographic technology, particularly high-frequency transducers and elastography modalities, have significantly improved diagnostic accuracy in leprosy-related neuropathy through enhanced spatial resolution and tissue characterization. The present results are also consistent with evidence that ultrasonography identifies subclinical neural involvement in seropositive household contacts, supporting integrated algorithms combining anti-PGL-1 serology and ultrasonography for surveillance and early diagnosis, especially when SSS test is negative and palpation is insensitive [11].

**Fig 2:**
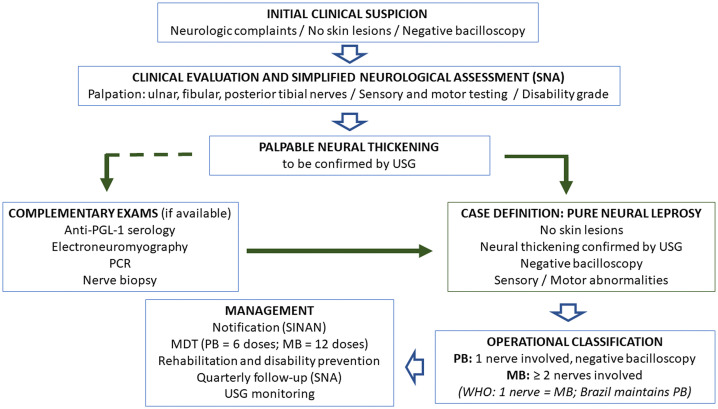
Proposed flowchart to support the diagnosis of pure neural leprosy.

Given the limitations of neural palpation, the issue of diagnostic accuracy in PNL emerges, since diagnosis in these cases relies solely on neurological semiology, strength, and sensation, while many other conditions cause peripheral neuropathy, even in endemic regions, and nerve biopsy remains difficult to access or perform [34,39,40]. Fig 2 presents a proposed flowchart to support the diagnosis of PNL.

It is essential to enhance the effectiveness of surveillance, diagnosis, and disability-prevention strategies, thereby contributing directly to global leprosy elimination goals. The generalization of our findings highlights diagnostic and operational classification failures when based solely on neurological palpation, particularly in pure neural cases, which may represent up to 10% of annual diagnoses in Brazil.

Taken together with the broader literature, our results suggest that reliance on neural palpation alone may be insufficient as a primary diagnostic approach [41,42]. The integration of ultrasonography, including portable systems, offers a more objective and reproducible approach to evaluating major peripheral nerves, particularly in resource-limited settings where early detection is crucial for preventing disability. Ultrasonography also enables evaluation of the median nerve, commonly affected in leprosy [16].

Median nerve enlargement was identified exclusively by ultrasonography in a substantial proportion of patients. In one case, this finding altered operational classification. Although this observation highlights the potential relevance of non-palpable nerves, it should be interpreted cautiously, given the small sample size and the possibility of alternative diagnoses, such as compressive neuropathies. Further studies are required to determine the clinical utility of systematic median nerve assessment in leprosy. Furthermore, bilateral asymmetry may serve as an early indicator of neural involvement [17].

Taken together, these aspects are important because median neuropathy may be missed clinically and may alter operational classification. However, because isolated median nerve enlargement at the wrist may also occur in carpal tunnel syndrome, its diagnostic value in leprosy lies mainly in its interpretation within a broader pattern of proximal involvement, asymmetry, and multinerve abnormalities [13,43]. Our findings therefore support the inclusion of median nerve ultrasonography in the evaluation of suspected neural leprosy, particularly in patients without skin lesions, and in endemic areas. Routine ultrasonographic assessment of the median nerve may also influence operational classification, potentially upgrading some patients with PNL to MB.

This study also showed that some patients with leprosy and neurological manifestations may not present nerve thickening detectable by ultrasonography, despite having functional disability (G1D/G2D). This suggests that nerve dysfunction may occur before significant structural enlargement becomes apparent. These findings are consistent with an Indian study of 40 patients, in which ultrasonography detected abnormalities in only 63% of cases [33].

Although important contributions have already been published in this field, future research should focus on standardizing ultrasonographic techniques applied to leprosy, establishing clear definitions of pathological nerve thickening, and advocating for their inclusion in national and international guidelines, thus bridging the gap between clinical practice and evolving diagnostic technologies [15,40]. For patients with difficult-to-diagnose neural manifestations, further investigation of electroneuromyography and molecular diagnostic techniques for leprosy would also be valuable.

The systematic study of nerve topography and cut-off values for defining peripheral nerve thickening is also important, as these vary considerably depending on the study population [15,20,21]. Higher-resolution ultrasonography may allow exploration of epineural features, nerve heterogeneity, and intraneural vascular flow, thereby improving diagnostic sensitivity [44].

This study has some considerations that help frame its results. Examinations were performed by three specialists (dermatology, orthopedics, physiotherapy) from a referral center in an endemic area, all using the same standardized palpation technique (identical hand positioning, bidigital rolling, graded pressure). Although differences in specialty background could introduce minor examiner effects, the shared protocol and setting support internal consistency and reflect routine practice. The single-center design and moderate sample size may limit the precision of some estimates, but procedures were uniform. Ultrasonography, our pragmatic reference test, was performed by a single, highly experienced operator based at the referral center, which enhances methodological consistency and likely reduces operator-related bias. The high prevalence of PNL in the sample reflected the role of referral centers in the diagnosis of atypical forms. Finally, electroneuromyography was not used to support the diagnosis of PNL; in line with BMH guidelines, we classified as PNL those patients with an exclusively neural presentation, peripheral nerve thickening accompanied by sensory, motor, and/or autonomic impairment in the corresponding territory, even in the absence of ancillary tests. This operational choice reflects endemic-area practice: these patients showed a clinical phenotype fully compatible with PNL, were close contacts of confirmed leprosy cases, and lived in a highly endemic setting, factors that increase pretest probability and support clinical classification when electrophysiology or other confirmatory tests are not readily available. Although a non-palpable nerve represents a distinct clinical finding from a normal nerve, these categories were merged because, in operational classification, both are considered non-affected. This analytical decision may have led to an overestimation of specificity.

This study has limitations. The sample size was relatively small and derived from a single referral center, which may limit generalizability. Ultrasonography was used as a comparative reference method rather than a definitive gold standard, and electrophysiological or histopathological confirmation was not systematically performed. In addition, multiple nerves were analyzed per patient, which may introduce clustering effects not fully accounted for in precision estimates. Finally, the cross-sectional design precludes evaluation of clinical outcomes or prognostic implications.

In conclusion, peripheral nerve palpation demonstrated limited sensitivity and specificity compared with ultrasonography, leading to errors in operational classification. Furthermore, interobserver agreement was very low, supporting the integration of ultrasonography as a complementary tool in diagnostic algorithms, particularly in clinically uncertain or pure neural cases, to enhance early detection of neural involvement, in alignment with the global leprosy elimination agenda.

## Supporting information

S1 Fig Representation of palpation of the radial (A), ulnar (B), fibular (C), and posterior tibial (D) nerves. (TIF)

S2 Fig Representation of ultrasonographic examination of the radial (A), ulnar (B), median (C), fibular (D), and posterior tibial (E) nerves. (TIFF)
