# Supplementary figures and images for: Diagnostic performance of peripheral nerve palpation compared with ultrasonography in leprosy neuropathy: A prospective real-world clinical evaluation
Source: PLoS Negl Trop Dis. 2026 Apr 21;20(4):e0014234. doi: 10.1371/journal.pntd.0014234 (PMC13143118; doi:10.1371/journal.pntd.0014234)

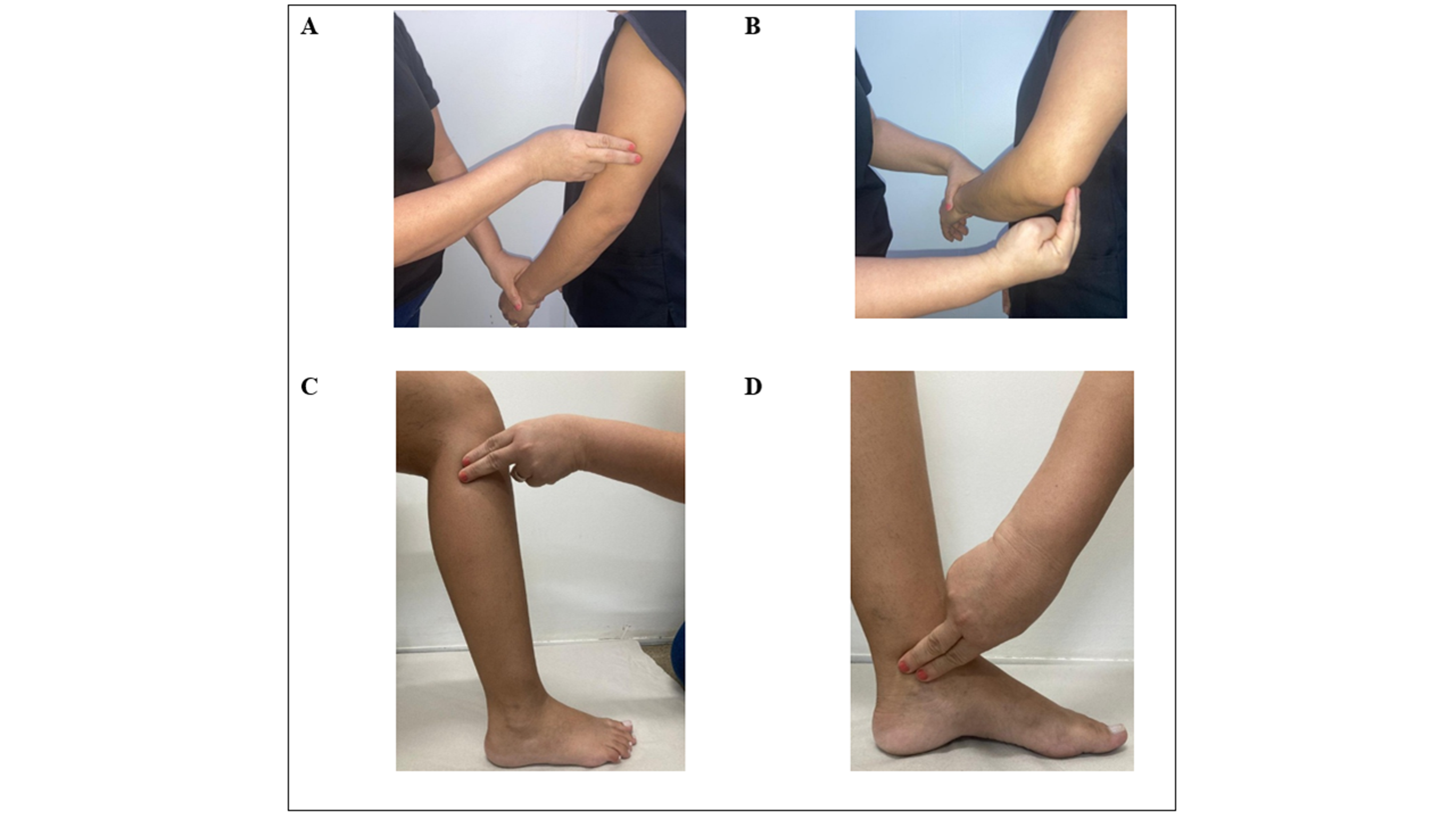

Supplement: S1 Fig — (TIF) [file pntd.0014234.s001.TIF]

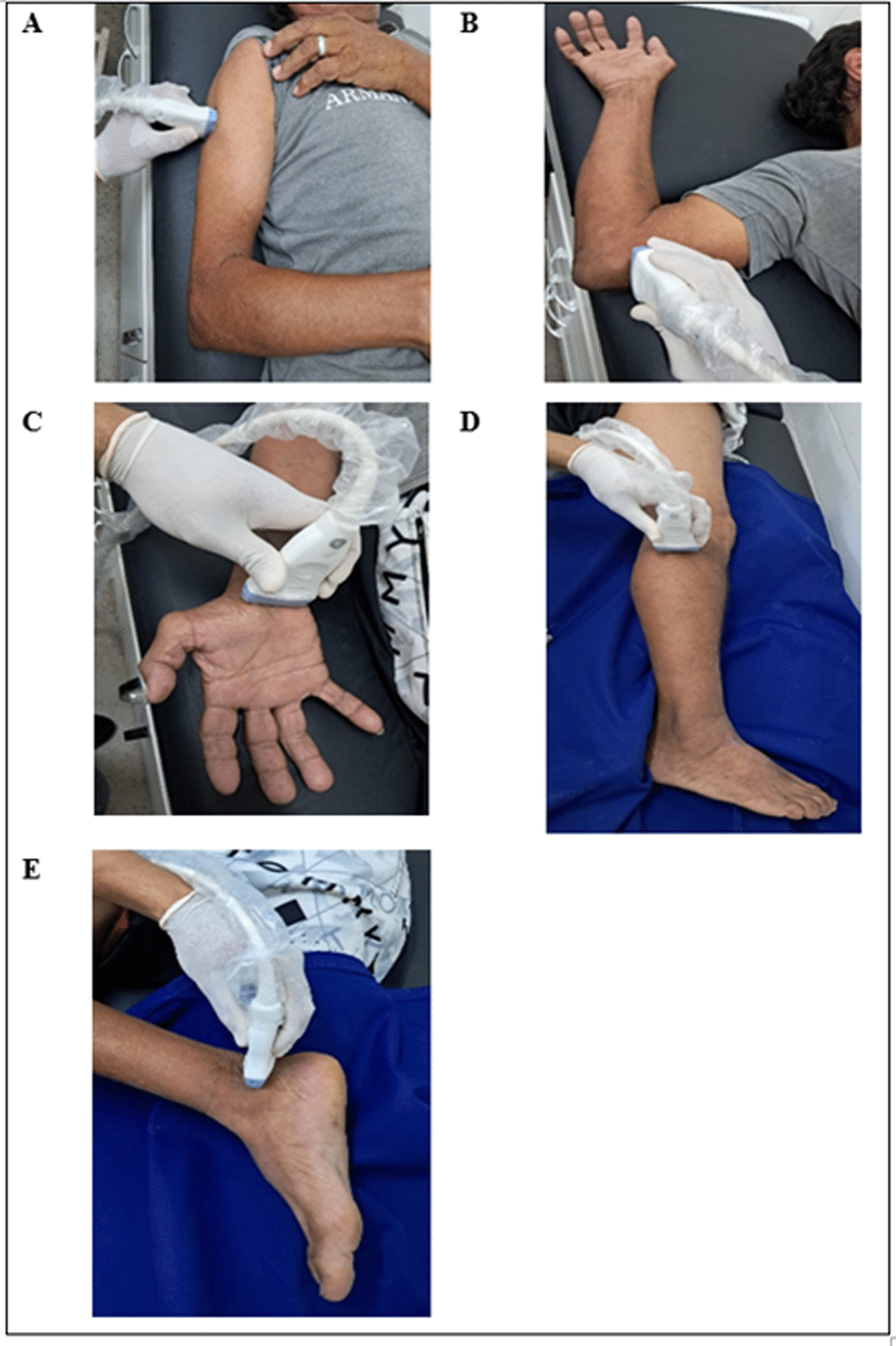

Supplement: S2 Fig — (TIFF) [file pntd.0014234.s002.tiff]
